# Microstructural Characterization of Defects and Secondary Phases in (Ti, Ta)C-Type Carbides in Nickel-Based Superalloys

**DOI:** 10.3390/ma19091875

**Published:** 2026-05-02

**Authors:** Xin Jin, Yunsong Zhao, Wei Chen, Pengfei Nan, Binghui Ge

**Affiliations:** 1 State Key Laboratory of Opto-Electronic Information Acquisition and Protection Technology, Leibniz International Joint Research Center of Materials Sciences of Anhui Province, Institutes of Physical Science and Information Technology, Anhui University, Hefei 230601, China; 15691421259@163.com (X.J.); chwei0929@163.com (W.C.); 2 Science and Technology on Advanced High Temperature Structural Materials Laboratory, Beijing Institute of Aeronautical Materials, Beijing 100095, China

**Keywords:** nickel-based superalloys, carbide, stacking faults, scanning transmission electron microscopy

## Abstract

**Highlights:**

- Metal carbides (MCs) with a core–shell structure were observed.
- A high density of stacking faults was observed within the MCs, and these stacking faults were located on the (001) plane.
- The precipitation of the M_23_C_6_ phase was observed at stacking faults within the MCs.

**Abstract:**

Metal carbides (MCs) serve as essential strengthening phases in nickel-based superalloys, so the decomposition of MCs during high-temperature creep is regarded as detrimental to the mechanical properties and service life of these alloys. However, detailed investigations of the MC decomposition process at the microscale remain limited. In this study, the microstructure of MCs (where M is a mixture of Ti and Ta) in a nickel-based superalloy was characterized using aberration-corrected scanning transmission electron microscopy. The MCs exhibit a spherical core–shell structure, with Ta enrichment in the shell and Ti segregation in the core. Moreover, a high density of Cr-rich stacking faults, accompanied by Cr-rich M_23_C_6_ precipitates at their terminations, was identified in the Ti-rich cores, suggesting that these defects may be closely associated with the decomposition of MCs. This study may further expand the fundamental understanding of the interactions between defects and carbide properties.

## 1. Introduction

Nickel-based superalloys, renowned for their exceptional high-temperature strength, creep resistance, and oxidation–corrosion resistance, are widely used in the production of high-temperature components such as aircraft engine turbine blades, gas turbine combustion chambers, and rocket engine nozzles [1,2,3,4,5]. In addition to the precipitation strengthening imparted by the precipitation of the γ’ phase in their inherent alloy microstructure, metal carbides, as one of the primary precipitates in nickel-based superalloys, also play a crucial role in determining their strength and plasticity [6,7,8,9,10,11].

Metal carbides, typically distributed within grains or along grain boundaries, effectively impede dislocation glide and strengthen the alloy by pinning these boundaries [12,13,14]. Compared to a granular morphology, a rod-like or sharp-angled MC more readily initiates micro-cracks within the alloy [15,16,17]. In addition, the addition of Ta has a significant effect on the composition and morphology of the primary MC, transforming coarse dendritic carbides into finer particles and increasing the hardness of both the MC and the matrix [18,19].

In fact, during the service of nickel-based superalloys, the microstructure is subjected to elevated temperatures and stress, leading to the decomposition of metal carbides. MCs and their decomposition product, M_23_C_6_, subsequently coarsen under these external conditions, consequently inducing the initiation and propagation of cracks [20,21,22,23]. Chemical decomposition is generally believed to initiate at the interface between the MC and the matrix, which is attributed to the common observation of M_23_C_6_ and γ’ phases forming around the MC decomposition zones [24]. Based on EDS and TEM analyses, most studies indicate that the decomposition proceeds via the following reactions: MC + γ → M_23_C_6_ + γ’ (in which M in M_23_C_6_ is predominantly Cr) and MC + γ → M_6_C + γ’ (in which M in M_6_C is primarily Mo and W) [25,26,27,28,29,30,31]. The decomposition of an MC induces degradation of the intra-granular and grain boundary microstructure, thereby leading to a reduction in the service life of the superalloy [32,33]. However, the detailed structural evolution at the early stages of this process, particularly the roles of crystallographic defects, local compositional fluctuations, and interfacial characteristics, has not yet been clearly and systematically understood.

Previous investigations into MC decomposition have primarily focused on the macroscopic scale, with limited reports addressing the process at the atomic level. In recent years, Ge et al. utilized aberration-corrected electron microscopy to discover that Cr segregates at dislocation cores, and the accompanying dislocation climb facilitates the formation of M_23_C_6_ precipitates along the dislocation lines, leading to MC decomposition [34]. Here, an advanced aberration-corrected microscopy technique is also used to further analyze the microstructure of MC in a nickel-based superalloy after extended exposure to high temperatures and stresses. A high density of Cr-rich stacking faults on the (001) planes was observed within the carbide, and Cr-rich M_23_C_6_ precipitates were identified at the termini of these stacking faults. These findings suggest a close correlation between the Cr-rich stacking faults and the decomposition of MC.

## 2. Materials and Methods

Nickel-based DD5 superalloy bars with the chemical composition listed in Table 1 were prepared by the Bridgman directional solidification method. The bars had dimensions of Φ 5 mm × 25 mm and were oriented along the [001] crystallographic direction in the axial direction. The alloy underwent a series of heat treatments, including heating at 1300 °C/2 h + 1120 °C/4 h + 1080 °C/4 h + 980 °C/4 h, followed by air cooling, then heating at 900 °C/4 h and air cooling. Subsequently, the specimens were exposed to 950 °C/235 MPa for approximately 60 h. After mechanical polishing to remove surface scratches, the surface was further treated using the Hitachi IM 4000II (Hitachi High-Tech Corporation, Tokyo, Japan). Ar ion milling was employed to flatten the surface and remove impurities, and its microstructure was examined via scanning electron microscopy (SEM). A TEM lamella was prepared, and backscattered-electron (BSE) imaging was conducted using a Carl Zeiss Crossbeam550L focused ion beam/scanning electron microscope (FIB-SEM) (Carl Zeiss AG, Oberkochen, Germany). Selected-area electron diffraction (SAED) analysis was performed on a JEM-F200 transmission electron microscope (JEOL Ltd., Tokyo, Japan) operated at 200 kV (TEM). High-angle annular dark-field scanning transmission electron microscopy (HAADF-STEM) imaging and energy-dispersive X-ray spectroscopy (EDS) were carried out on a Thermo Fisher Scientific Themis Z double spherical aberration-corrected electron microscope (Thermo Fisher Scientific, Waltham, MA, USA) operating at 300 kV, using a convergence semi-angle of 25 mrad and collection semi-angles of 50–200 mrad.

## 3. Results and Discussion

**Figure 1:**
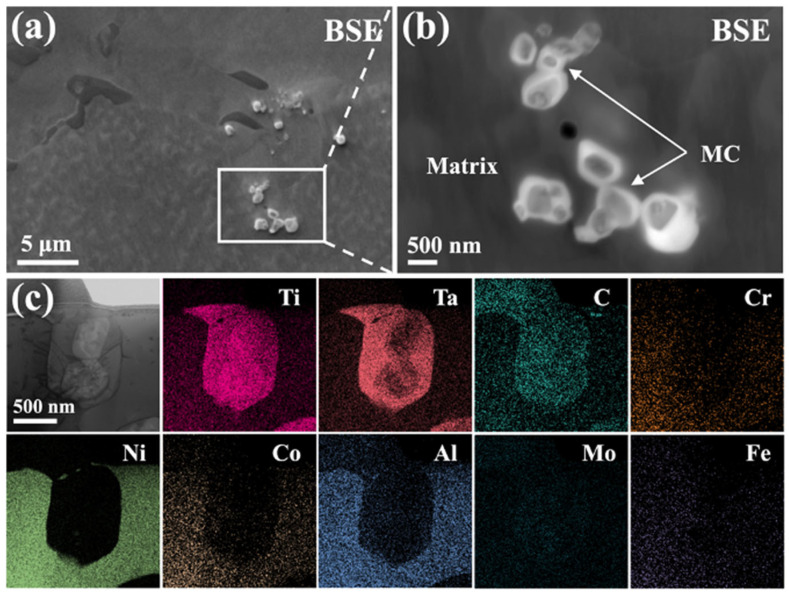
Morphology and compositional analysis of precipitates in the nickel-based superalloy. (**a**) Backscattered electron (BSE) image of the nickel-based superalloy, (**b**) BSE image showing the precipitates at higher magnification, (**c**) TEM lamella sample prepared from the precipitate and the corresponding energy-dispersive X-ray spectroscopy (EDS) elemental mappings.

Figure 1a,b show backscattered electron (BSE) images of the nickel-based superalloy, in which bright precipitates exhibit a distinct contrast difference between the core and shell regions. Specifically, the core appears darker, whereas the shell is brighter, indicating a core–shell structure. According to the imaging principle of BSE, the brighter outer region suggests enrichment in heavier elements, while the darker core is mainly composed of relatively lighter elements. This interpretation is further supported by the EDS results in Figure 1c, which show that the precipitates are mainly composed of Ti, Ta, and C. Specifically, the core is richer in Ti, while the shell is enriched in Ta, in agreement with the BSE observations. Based on the EDS results, these precipitates are inferred to be MC, either TiC or TaC [35].

**Figure 2:**
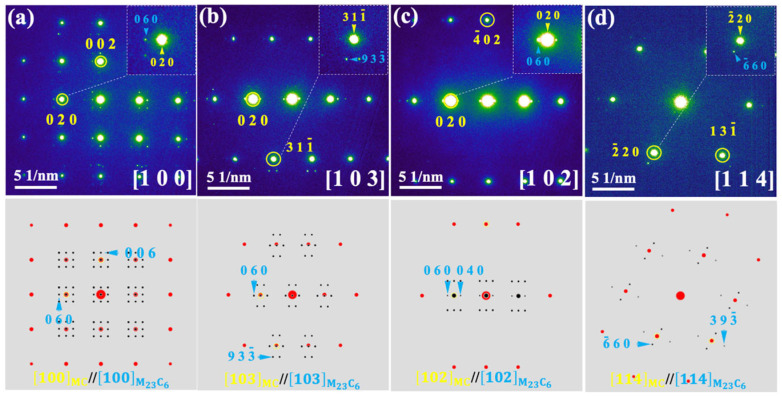
Electron diffraction analysis of the precipitate. (**a**–**d**) Experimental SAED patterns and simulation images. The boxes indicate the enlarged views of the corresponding diffraction spots.

To further identify the precipitates, selected-area electron diffraction (SAED) analysis is conducted. Diffraction patterns are acquired along four different zone axes, as shown in Figure 2a–d. By indexing the primary diffraction spots marked in yellow and combining these results with the EDS elemental maps in Figure 1c, the precipitates are identified as MC (where M is a mixture of Ti and Ta), with a cubic structure and a space group of Fm-3m. Furthermore, the presence of weaker diffraction spots (marked in blue) adjacent to the primary yellow ones in Figure 2a–d suggests the possible existence of a secondary phase within the MC.

**Figure 3:**
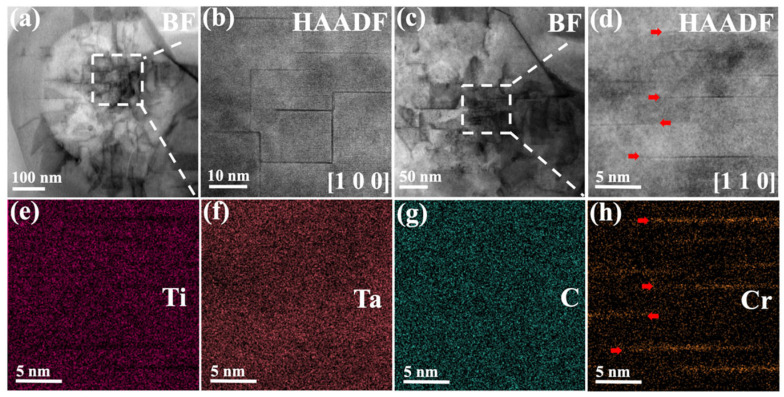
Characterization of defects and chemical composition within the MC. (**a**,**b**) Defect morphology viewed along the [100] zone axis and (**c**,**d**) along the [110] zone axis. (**e**–**h**) EDS elemental mappings of Figure 3d acquired from the [110] zone axis. The defects indicated by the red arrows in Figure 3d corresponds to the defects indicated in Figure 3h.

Moreover, to further reveal the internal structure of the MC, detailed characterization was carried out using HAADF-STEM. As shown in Figure 3a,c, in the low-magnification BF image, a high density of linear defects is present within the carbide. When observed along the [100] zone axis (Figure 3b), these defects appear as two sets of orthogonally arranged linear defect arrays are observed. In contrast, only a single set of defects is visible in the [110] orientation (Figure 3d). In addition, the EDS elemental maps (Figure 3e–h) of the defect regions in Figure 3d reveals significant Cr segregation at these sites.

**Figure 4:**
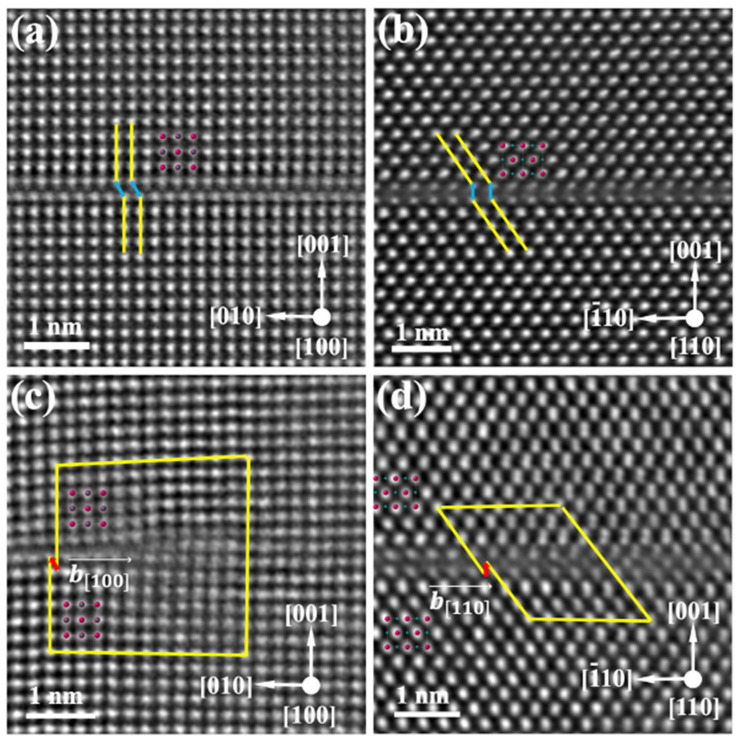
Analysis of stacking-fault structures and terminating partial dislocations in MC. (**a**,**b**) HAADF-STEM images of the stacking faults viewed along the [100] and [110] zone axes, respectively. (**c**,**d**) HAADF-STEM images of the partial dislocations at the stacking-fault termini, viewed along the [100] and [110] zone axes, respectively.

High-magnification HAADF images acquired along the [100] and [110] zone axes (Figure 4a,b) identify these defects as stacking faults located on the (001) plane. HAADF-STEM images of the partial dislocations at the stacking-fault termini are shown in Figure 4c,d along the [100] and [110] projections, respectively. Burgers circuit analysis determines the projected Burgers vectors, $\vec{b_{\left[100\right]}}$ and $\vec{b_{\left[110\right]}}$, to be 1/4[012] and 1/2[001], respectively. Since HAADF-STEM imaging provides only the two-dimensional projection of the Burgers vector, the results from the two viewing directions are combined to determine the true three-dimensional Burgers vector. The projected vector $\vec{b_{\left[100\right]}}$ can be geometrically decomposed into components along the [010] and [001] directions, expressed as

(1) $$\vec{b_{\left[100\right]}}=\frac{1}{4}\left[012\right]=\frac{1}{4}\left[010\right]+\frac{1}{2}\left[001\right]$$

Therefore, the Burgers vector component along the [001] direction is consistently determined to be 1/2[001] from both zone axes. In the [100] zone axis, the Burgers vector has a component of 1/4[010] along the [010] direction (Figure 4c), whereas in the [110] zone axis, no component is observed along the [$\bar{1}$10] direction (Figure 4d). This indicates that the component vector lies along the [110] direction with a magnitude of 1/4[110]. The spatial Burgers vector is thus determined as

(2) $$\vec{b}=\frac{1}{4}\left[110\right]+\frac{1}{2}\left[001\right]=\frac{1}{4}\left[112\right]$$

**Figure 5:**
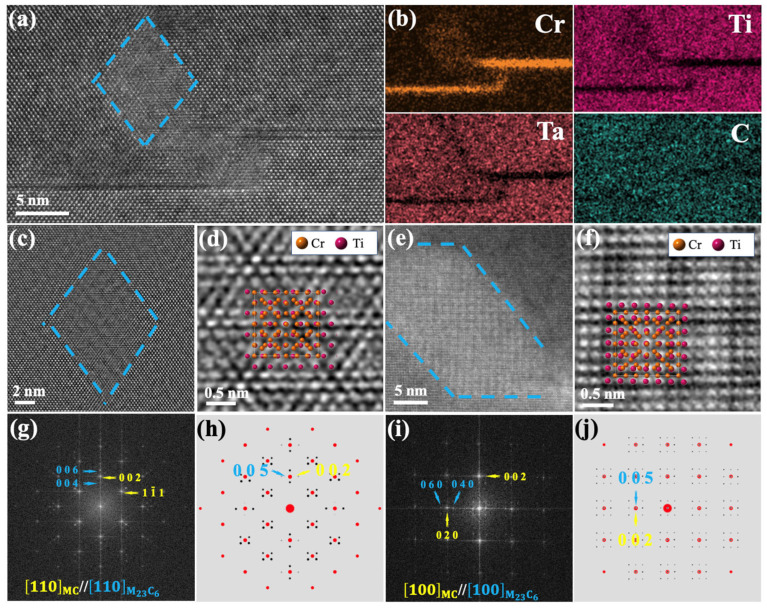
Elemental distribution and atomic structure analysis of the MC/M_23_C_6_ interface. (**a**) HAADF-STEM image of the MC/M_23_C_6_ interface. (**b**) EDS elemental mappings of the MC/M_23_C_6_ interface and the stacking-fault region. (**c**,**d**) HAADF-STEM images of the MC/M_23_C_6_ interface viewed along the [100] zone axis and (**e**,**f**) along the [110] zone axis. (**g**,**h**) FFT pattern from Figure 5c and the corresponding simulated pattern, and (**i**,**j**) FFT pattern from Figure 5e and the corresponding simulated pattern.

**Figure 6:**
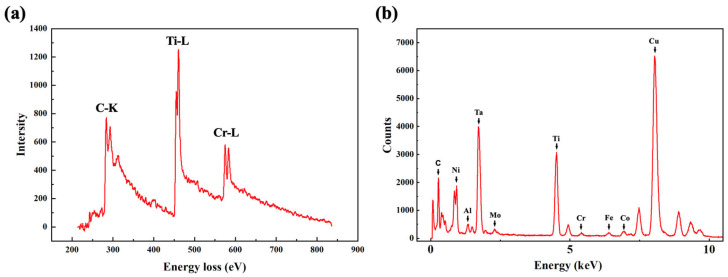
(**a**) EELS analysis of the contrast-anomalous region in the MC. (**b**) EDS elemental spectrum of the MC.

It should be noted that a region with anomalous contrast is observed near the partial dislocation at the stacking-fault terminus, as shown in Figure 5a. EDS elemental analysis of this region and the adjacent stacking-fault area, presented in Figure 5b, reveals a significantly higher Cr concentration at these locations. High-resolution images acquired from this area along the [100] and [110] zone axes exhibit a similar long-period contrast feature (Figure 5c–f). Fast Fourier Transform (FFT) analysis (Figure 5g,i) reveals the presence of satellite diffraction spots surrounding the primary reflections of the MC. Previous studies have suggested that the satellite diffraction spots surrounding the main reflections may also originate from carbonitrides, such as Ti(C, N) [36]. To exclude this possibility, EELS analysis was performed on the anomalous contrast region within the MC, and EDS spectrum analysis was also conducted on the MC. As shown in Figure 6, the anomalous contrast region is mainly composed of Ti, C, and Cr. In contrast, the edge energy of N is expected at 401 eV, but no obvious edge onset is observed at this position in the corresponding EELS spectrum. Therefore, the possibility that the observed feature is caused by carbonitrides can be excluded. According to the existing literature, MCs can decompose during service, primarily forming M_23_C_6_ or M_6_C phases [7,37]. By correlation of these observations with the SAED results (Figure 2) and diffraction simulations (Figure 5h,j), we identify these additional spots as originating from a secondary phase, namely M_23_C_6_. The anomalous contrast is attributed to moiré fringes formed between the M_23_C_6_ precipitates and the MC matrix. The lattice parameter of M_23_C_6_ is approximately 2.5 times that of the MC. Consequently, for both the [100] and [110] zone axes, the diffraction vectors g(002) MC//g(005) M_23_C_6_ and g(020) MC//g(050) M_23_C_6_ share identical interplanar spacings [34]. In summary, the combination of EDS, HAADF-STEM imaging, diffraction patterns, and FFT analyses confirms the precipitation of Cr-rich M_23_C_6_ within the MC.

**Figure 7:**
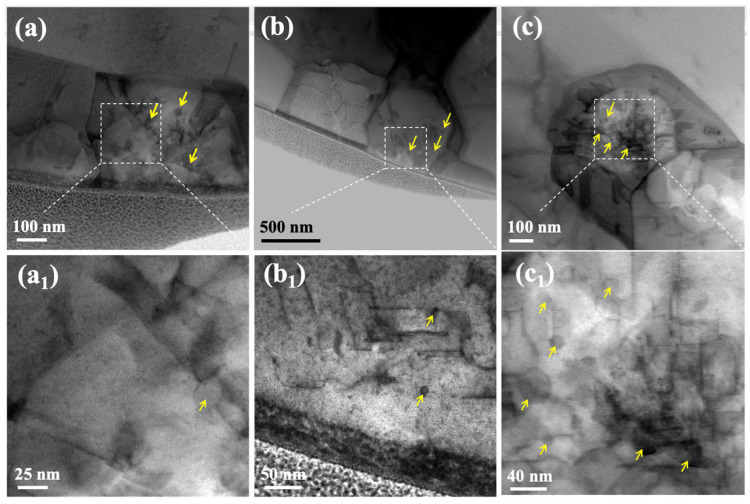
Low-magnification BF images of three selected MCs and their corresponding high-magnification BF images. (**a_1_**), (**b_1_**), and (**c_1_**) are high-magnification BF images of the boxed regions in (**a**), (**b**), and (**c**), respectively. The yellow arrows indicate the distribution of the M_23_C_6_ phases.

**Figure 8:**
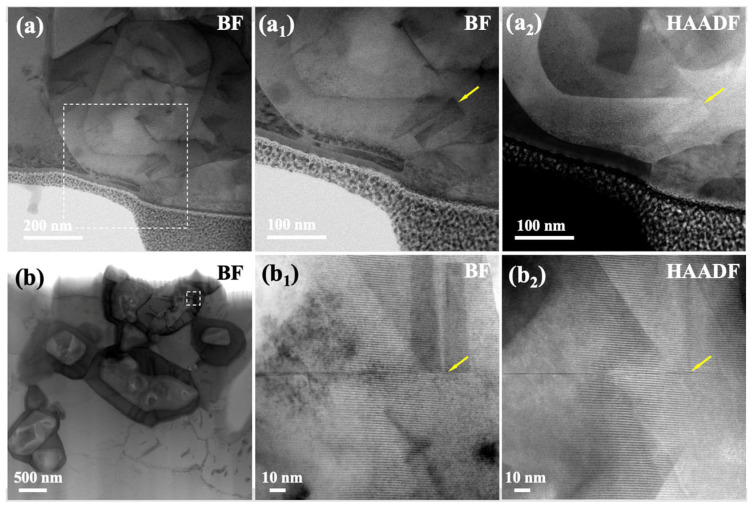
Low-magnification BF images of three selected MCs and their corresponding high-magnification BF and HAADF images. (**a_1_**) and (**a_2_**) are the high-magnification BF and HAADF images of the boxed region in (**a**), respectively. (**b_1_**) and (**b_2_**) are the high-magnification BF and HAADF images of the boxed region in (**b**), respectively. The yellow arrows indicate the stacking faults.

Furthermore, the nickel-based superalloy studied here contains Ta and Ti. These elements partition within the carbide precipitate, with Ta predominantly located in the outer shell and Ti in the core, forming a distinct core–shell structure. Notably, as shown in Figure 7, the number of stacking faults in the outer shell region of the carbide is significantly lower than that in the core region, and no M_23_C_6_ phase is observed in the outer shell. Combined with the results presented in Figure 8 and Table 2, it can be concluded that the internal region of the MC contains more defects than the external region, and that M_23_C_6_ is observed only in the internal region. According to the reported thermochemical data, the bond dissociation energies D_0_(MC) of TiC and TaC are 3.857(4) and 4.975(3) eV, respectively, while the corresponding diatomic enthalpies of formation, ${∆}_{f}H{}^{\circ}(MC(g))$, are 810.0(16.7) and 1012.6(2.2) $kJ\;{mol}^{-1}$, respectively [38]. Compared with TiC, TaC exhibits both a higher M-C bond dissociation energy and a larger enthalpy of formation, indicating stronger Ta-C bonding and higher thermochemical stability. This suggests that, under thermal or external perturbations, bond rupture in TaC requires a higher energy input, and therefore the dissociation and decomposition of TaC are energetically less favorable than those of TiC. From this perspective, TaC is expected to be more resistant to structural degradation associated with local bond breaking, carbon loss, or phase transformation. These results may indicate that Ta suppresses the formation of stacking faults, thereby inhibiting the transformation of MC into M_23_C_6_ and consequently enhancing the creep resistance of the alloy.

## 4. Conclusions

Aberration-corrected scanning transmission electron microscopy is employed to characterize MCs in a nickel-based superalloy after high-temperature creep. This investigation reveals a core–shell architecture within the carbides, featuring a Ta-rich shell and a Ti-rich core. Microstructural analysis further uncovers a high density of basal-plane stacking faults inside the MCs. The partial dislocations at the fault termini exhibit a Burgers vector of a/4[112]. Notably, M_23_C_6_ precipitates at these stacking-fault ends, with significant Cr enrichment detected both within the precipitates and along the faults. This suggests that Cr-rich stacking faults serve as critical sites for facilitating the transformation of MC to M_23_C_6_. Moreover, a marked contrast is observed between the core and shell regions: the Ta-rich shell contains considerably fewer stacking faults and shows no M_23_C_6_ precipitation, whereas the Ti-rich core contains numerous stacking faults associated with M_23_C_6_ formation. This indicates that Ta may suppress stacking-fault formation, thereby inhibiting the MC-to-M_23_C_6_ transformation.

## Figures and Tables

**Table 1:** Chemical composition of the alloy in this work (wt.%).

B	C	Cr	Co	Al	Mo	W	Ta	Ti	Ni
0.023	0.16	15.7	15.7	5.2	3.02	0.71	0.8	2.4	Bal.

**Table 2:** Numbers of stacking faults and M_23_C_6_ phases in the internal and external regions of the three MCs in Figure 7a–c.

	(a)	(b)	(c)
the number of internal stacking faults	12	18	32
the number of external stacking faults	3	4	11
the number of internal M_23_C_6_ phases	0	0	0
the number of external M_23_C_6_ phases	3	3	7

## Data Availability

The original contributions presented in this study are included in the article. Further inquiries can be directed to the corresponding authors.
